# Management of retro-acetabular osteolysis with a retained shell and injection of demineralized bone matrix using a vinyl urinary catheter and syringe: a simple technique and case series

**DOI:** 10.1186/s42836-025-00349-4

**Published:** 2025-12-04

**Authors:** Rit Apinyankul, Lelyn Lindo Silva, Puthi Tantikosol, Stuart B. Goodman

**Affiliations:** 1 Department of Orthopaedics, Faculty of Medicine, Khon Kaen University, Khon Kaen, 40002 Thailand; 2 Department of Nursing, Stanford Health Care, Stanford, CA 94305 USA; 3 Institute of Orthopaedics, College of Medicine, Lerdsin General Hospital, Rangsit University, Bangkok, 10500 Thailand; 4 Department of Orthopaedic Surgery, Medical Center Outpatient Center, Stanford University, Stanford University School of Medicine, Stanford, CA 94305 USA

**Keywords:** Retro-acetabular, Osteolysis, Vinyl urinary catheter, Demineralized bone matrix, Injection technique

## Abstract

**Background:**

The management of retro-acetabular osteolysis in revision hip arthroplasty with acetabular component retention remains controversial and challenging due to limited access to the area.

**Surgical technique and methods:**

Fourteen patients with well-fixed and well-aligned acetabular components underwent revision surgery and a retained shell. A vinyl urinary catheter and syringe were used to deliver demineralized bone matrix putty to the bone defect after debridement. Clinical outcome and radiographic follow-up were scheduled at a minimum of 2 years.

**Results:**

The revision arthroplasty survivorship rate with this technique was 85.7% (12 of 14 patients) at a median follow-up of 6 years. One cup failed due to an aseptically loosening cup, and another from late septic loosening. Significant improvement of the University of California Los Angeles (UCLA) score, Harris Hip Score (HHS) pain subscale, and Hip Disability and Osteoarthritis Outcome Score for Joint Replacement (HOOS JR) were observed at a median 6-year follow-up.

**Conclusions:**

Management of retro-acetabular osteolysis with injected demineralized bone matrix using a syringe and vinyl urinary catheter is a reliable, easy, low-cost method with satisfactory mid-term clinical outcome improvement.

Video Abstract

**Supplementary Information:**

The online version contains supplementary material available at 10.1186/s42836-025-00349-4.

## Introduction

Given the success of primary total hip arthroplasty (THA) and advanced prosthesis designs, hip joint reconstruction continues to increase in not only lower demand, but also higher demand younger patients [1, 2]. The incidence of revision THA continues to rise, and is projected to increase by 43–137%% in 2030 [3, 4]. Aseptic loosening is a common cause of THA failure (4–11%) [5, 6] and related to periprosthetic osteolysis due to wear debris. Revision surgery is also indicated if there is progressive wear, impending wear-through, or rapidly progressive osteolysis [7–9]. Treatment aims to stop the progression of periacetabular osteolysis with (a) revision of a well-fixed cup, which may result in further acetabular bone loss, or (b) cup retention with bone defect management. Treatment options for periacetabular osteolysis with a well-fixed and well-aligned acetabular component are available with variable outcomes [10–13]; Type I and II American Academy of Orthopaedic Surgeons (AAOS) acetabular deficiencies can be managed with particulate bone graft and/or bone substitute [14]. The osteolytic area may be accessed for debridement and bone grafting through empty screw holes or by cortical windows; some studies showed regression of osteolytic lesions regardless of performing debridement and/or bone grafting [15–17]. Modular cup retention with liner and femoral head exchange has the advantage of a low risk of reoperation and low cost; however, bone graft/substitute incorporation is challenging due to limitations of accessibility to the osteolytic area. Full acetabular component revision jeopardizes bone stock and fixation and is associated with a higher reoperation rate [18].

This study aims to introduce an easy, reproducible, and low-cost technique for grafting retro-acetabular osteolysis in cases where the modular shell is stable and well-positioned.

## Methods

This cohort study received ethical approval, including informed consent from participants, granted by the Institutional Review Board at Stanford University, Stanford, California, USA (Approval No.10669).

We reviewed 14 revision THA cases (January 2009–September 2020) from our arthritis service joint registry in which acetabular shell retention and demineralized bone matrix (DBM) putty grafting were used to manage retro-acetabular bone loss during revision THA. All patients had radiographic evidence of periacetabular osteolytic lesions around a cementless cup with impending or existing failure of a polyethylene liner, but without any sign of shell loosening or migration. Eight of the fourteen patients underwent computed tomography (CT) scans to assess the severity of retro-acetabular bone defects, all of which showed retro-acetabular osteolytic areas involving less than 50% of the host bone–cup contact area. The remaining six patients had minimal retro-acetabular osteolysis on plain radiographs, obviating the need for CT evaluation. All 14 patients had a minimum of 2 years of clinical and radiographic follow-up.

### Equipment

**Fig. 1:**
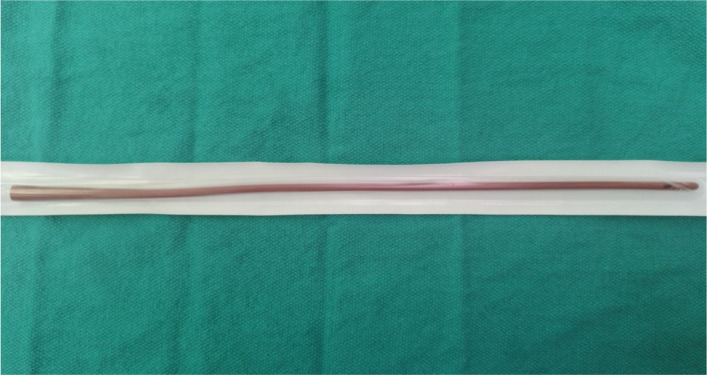
Dover™ Rob-Nel latex-free Catheter, 16″ length, smooth rounded tip, 16 Fr (5.3 mm)

1. Demineralized bone matrix putty in 10 cc. syringe (DBX® Putty, 038100, DePuy Synthes, 1302 Wrights Lane East, West Chester, PA 19380, USA) processed by Musculoskeletal Transplant Foundation, 125 May Street, Edison, NJ 08837, USA
2. Dover™ Rob-Nel latex-free Catheter (Fig. 1), 16″ length, smooth rounded tip, 16 Fr (5.3 mm, 8888492058, Covidien™, Covidien LLC, 15 Hampshire Street, Mansfield, MA 02048 USA)
3. 10 cc. normal saline-loaded syringe for injection and irrigation through screw holes

### Surgical technique

Acetabular exposure must be obtained circumferentially with removal of wear debris and all peri-acetabular fibrous tissue to assess cup stability. Well-fixed polyethylene liner inserts are removed by a drill and screw technique, taking care not to damage the shell surface and locking mechanism. After the polyethylene liner was disengaged from the locking mechanism, all screws were removed. The shell stability was then assessed by applying an eccentric force to the edge while observing for blood or fluid at the bone-shell interface [19, 20]. In cases where some acetabular screws cannot be removed for any reason (e.g., stripped or damaged screws), these screws may be left in place, and only partial removal is performed after acetabular shell stability is confirmed. Locations of osteolysis should be identified preoperatively. Candidates for cup retention should have an acetabular component with the following characteristics (a) a good clinical track record, (b) be well-fixed and well-positioned after all screws are removed, (c) have a large enough cup size to accommodate an available new liner with appropriate femoral head size for hip stability, and (d) stable locking of the new liner and good intra-operative hip stability [19, 21–23].

If the acetabular component is found to be well fixed and well positioned, then.

**Fig. 2:**
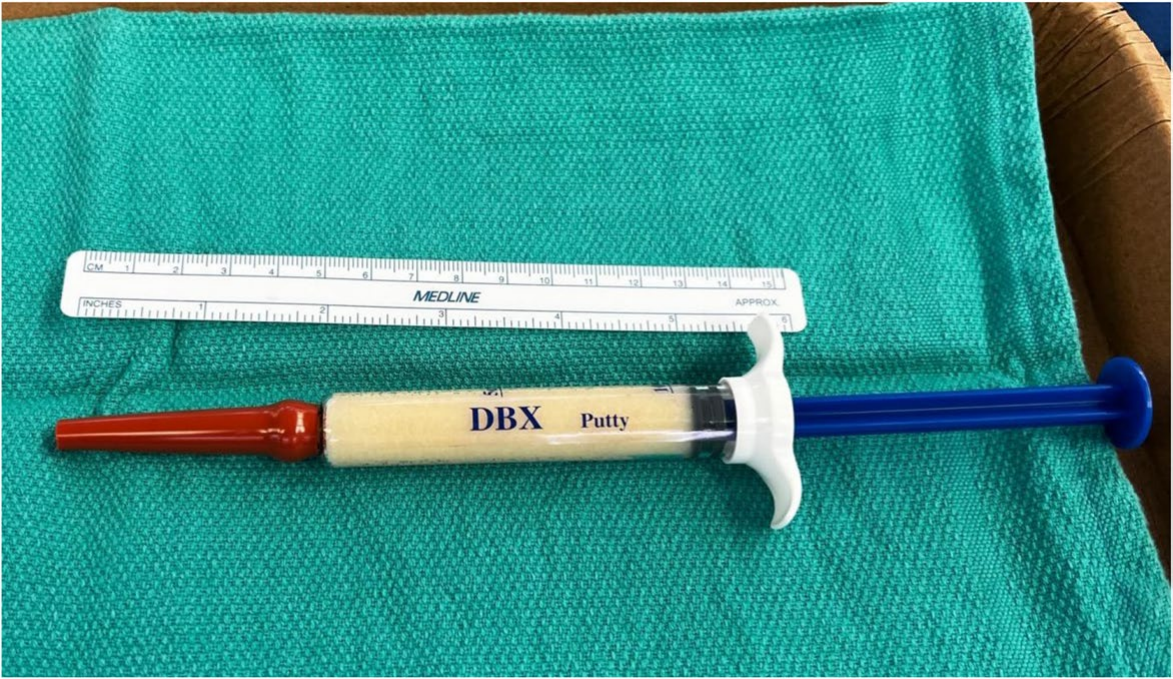
Demineralized bone matrix putty in 10 cc. syringe (DBX® Putty) integrated with 2-inch sterile vinyl urethral catheter

1. The osteolytic area is debrided via acetabular screw holes with varying-angle small bone curettes.
2. A ten-milliliter normal saline-loaded syringe integrated with a vinyl urinary catheter is used for irrigation through the screw holes repeatedly until no debris is seen coming from the holes.
3. We used DBM putty to function as an osteoconductive scaffold with potential inductive properties to help restore retro-acetabular bone stock and prevent the progression of osteolysis. The DBM putty 10 cc. The syringe is connected to 2 inches of a vinyl urinary catheter to deliver the DBM into the acetabular defect. After cutting the urethral insertion side off, 2 inches of the funnel side of the catheter was firmly connected to the syringe (Fig. 2). This allows very easy passage of a 5.3 mm diameter catheter through the 6.5 mm screw holes of the acetabular cup, which could be directed to different directions.
4. When the tip of the catheter reaches the bottom of the bone defect, the surgeon starts injecting DBM putty and feels push-back pressure during bone defect filling up (like bone cement application by cement gun). A longer catheter can be applied to the syringe in cases of deep screws holes or difficult access in obese patients.
5. The amount of applied DBM putty is dependent on the extent of the retro-acetabular defect, and ranges from 6–20 cc.
6. The surgeon may close other screw holes by fingertip technique or insert another catheter to prevent DBM leak during pressure application to the DBM putty syringe.

Then, the new liner is inserted with a well-functioning locking mechanism and head size. Cementing a new polyethylene liner into the acetabular component may be used with documented durable mid-term fixation [7, 10, 23].

### Imaging assessment

The indication for computed tomography (CT) was based on radiographic findings and clinical judgment. Eight patients underwent CT for further quantification when plain radiographs suggested lesions involving ≥ 25% of the cup–bone interface or when the lesion extent was uncertain. For six patients with small, well-delineated lesions on radiographs, CT was deemed unnecessary as it was not expected to alter management. We have explicitly addressed in the Limitations section that this protocol may have led to an underestimation of osteolysis extent and introduced a bias toward more favorable remodeling outcomes.

All patients were assessed clinically and radiographically at 3, 6, and 12 months, then every 2–3 years. We gathered implant information (type of cup and polyethylene liner, preop and postop head size, retained cup alignment, perioperative and postoperative complications, and cause of revision THA failure). The patient-reported outcomes (University of California Los Angeles (UCLA) score, Veterans RAND 12 (VR-12) physical subscale, VR-12 mental subscale, Harris Hip Score (HHS) pain subscale, HHS function subscale, and Hip Disability and Osteoarthritis Outcome Score for Joint Replacement (HOOS JR)) were retrieved from our database recording system.

### Statistical analysis

Descriptive statistics are presented as frequencies and percentages for categorical variables. For continuous variables, both parametric (means, 95% confidence intervals (CI), and standard deviations) and non-parametric (medians, interquartile ranges (IQR), and ranges) descriptive statistics are presented. Normality was assessed using the Shapiro–Wilk test. All patient-reported outcomes were tested for significant difference with a paired *t*-test for normally distributed data and a Wilcoxon signed rank test for non-normal data. All analyses were performed with SAS version 9.4 (Cary, NC, USA) with a two-sided level of significance of *α* = 0.05.

## Results

**Fig. 3:**
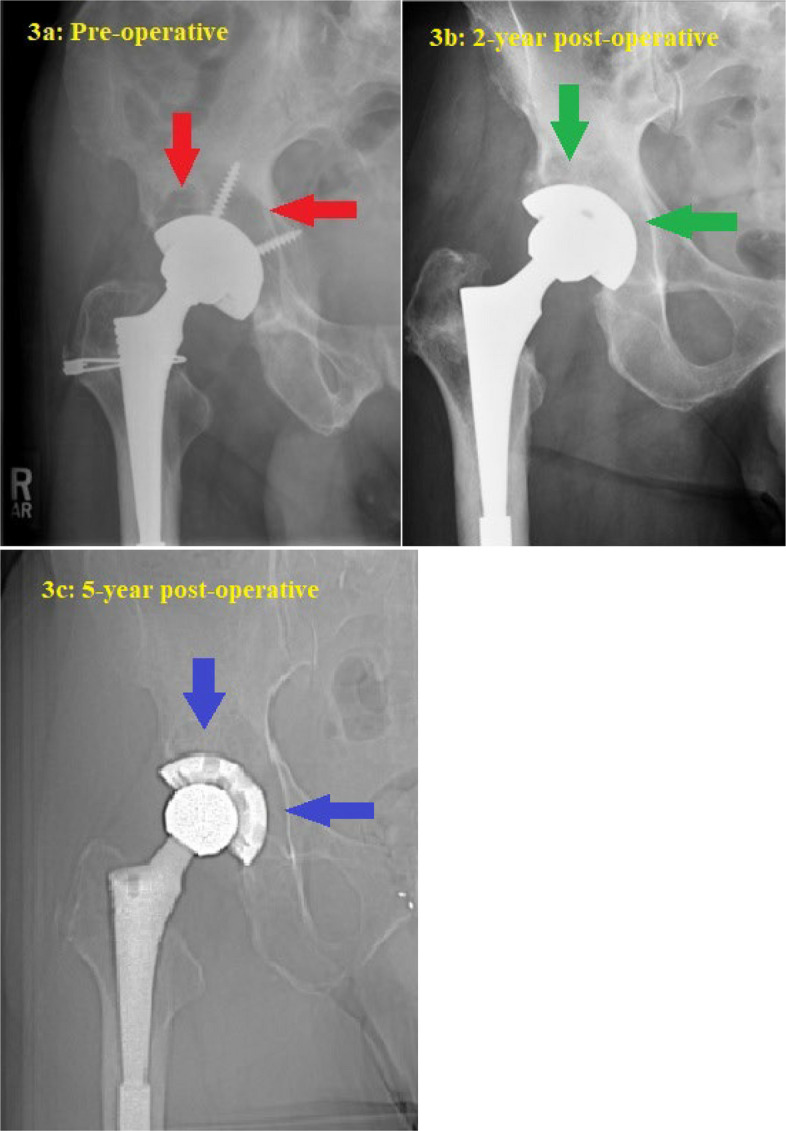
Serial imaging of a 74-year-old male following isolated liner and head exchange for retro-acetabular osteolysis, with retention of the well-fixed cup and stem. All screws were removed for stability testing and to access the retro-acetabular region for grafting with demineralized bone matrix using a vinyl urinary catheter and syringe. **a** Pre-operative radiograph showing retro-acetabular osteolysis (red arrows); **b** Two-year post-operative radiograph demonstrating the incorporation of demineralized bone matrix (green arrows) in the previous osteolytic lesion; **c** Five-year post-operative CT scout view revealing near-complete resolution of the retro-acetabular osteolysis (blue arrows)

**Fig. 4:**
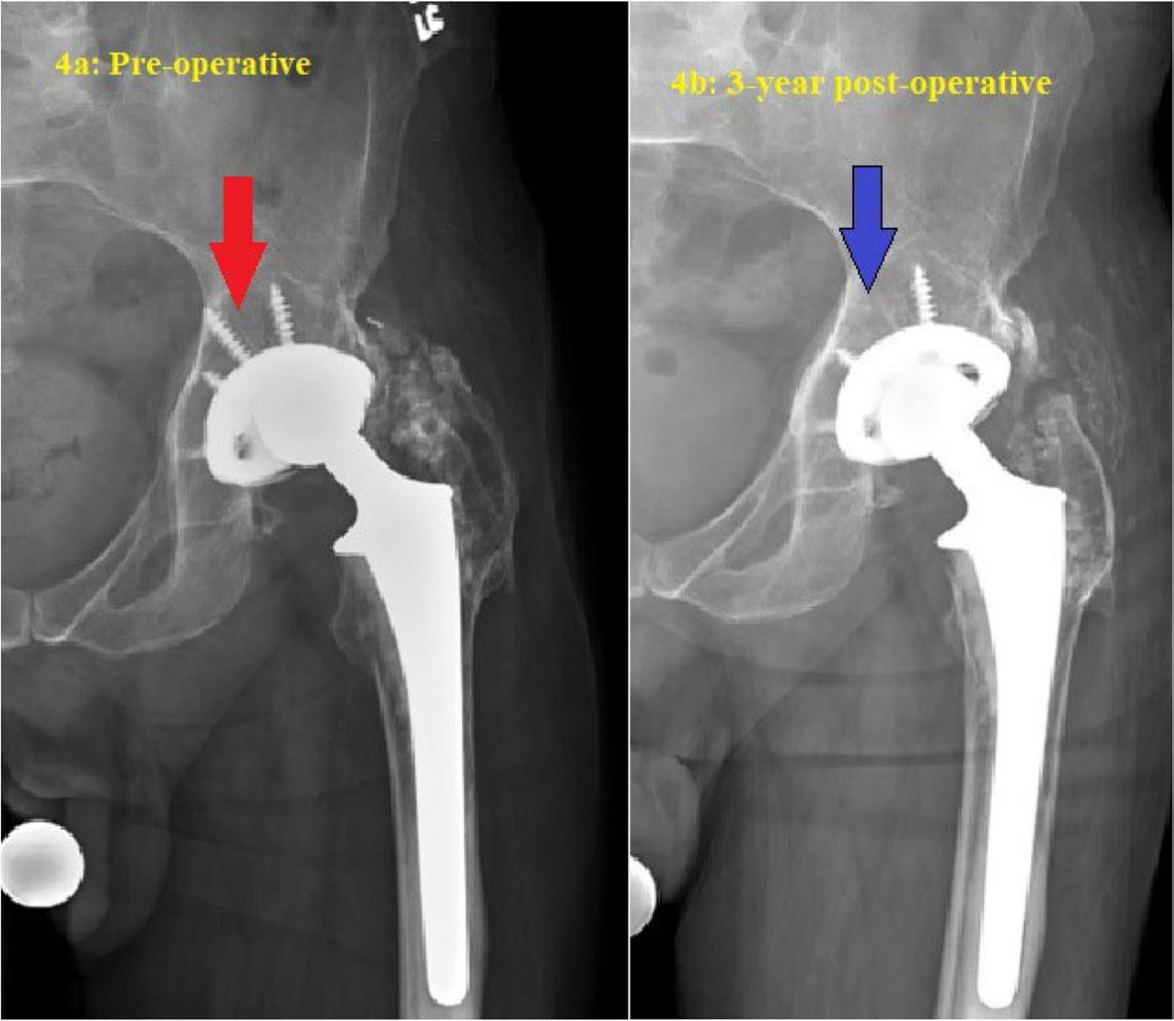
Serial imaging of an 80-year-old male following isolated liner and head exchange for retro-acetabular osteolysis secondary to polyethylene wear. The well-fixed cup and stem were retained. A single screw was removed to access the retro-acetabular region for grafting with demineralized bone matrix using a vinyl urinary catheter and syringe. **a** Pre-operative radiograph showing retro-acetabular osteolysis around the screw (red arrows); **b** Three-year post-operative radiograph demonstrating the incorporation of demineralized bone matrix (blue arrows) in the previous osteolytic lesion

**Fig. 5:**
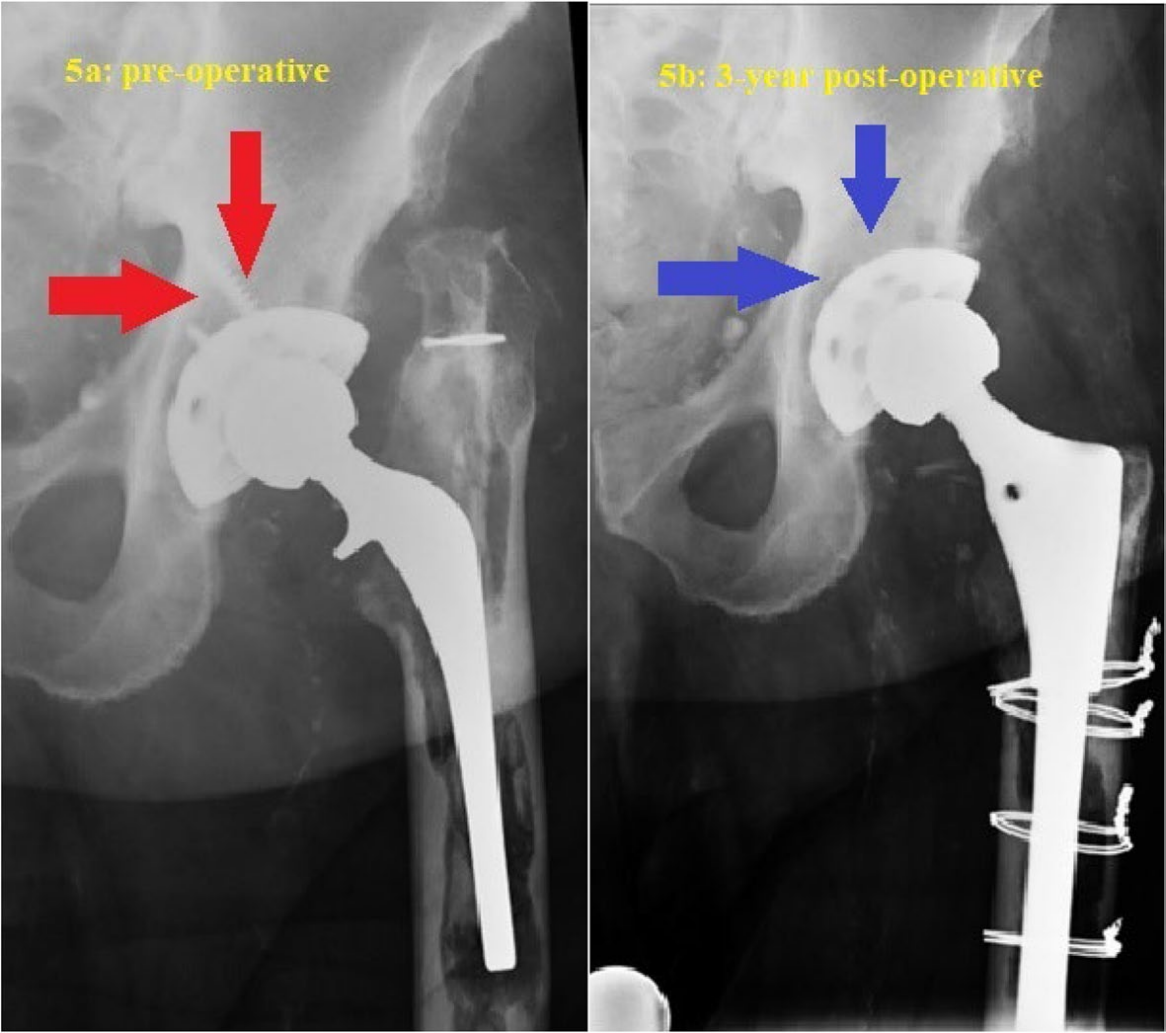
Serial imaging of an 83-year-old male after revision arthroplasty for a loose femoral stem and polyethylene wear. The well-fixed cup was retained, and all screws were removed for stability testing and to allow for retro-acetabular grafting. Demineralized bone matrix was delivered via a syringe and a vinyl urinary catheter. **a** Pre-operative radiograph showing retro-acetabular osteolysis around the screw (red arrows); **b** Three-year post-operative radiograph revealing the incorporation of demineralized bone matrix (blue arrows) in the previous osteolytic lesion

Fourteen patients underwent the above technique. Baseline patient characteristics and implant information are summarized in Tables 1 and 2, respectively. All polyethylene inserts were revised to highly cross-linked polyethylene (HXLPE) liners with appropriate head size for optimal stability and range of motion. Cementing new liners into retained well-fixed cups was performed in two patients with failed locking mechanisms. Median volume of DBM putty used for retro-acetabular bone defect was 10 cc (range 6–20 cc.). The mean cup inclination and anteversion were 44.5 ± 6.6 degrees and 26.6 ± 9.2, respectively. Median follow-up time was 71.5 months (range 19–140 months). Two of fourteen patients (14.3%) had failure of their THA revision. The first failure was due to aseptic loosening of the cup after a fall at 2 years post-op. The second revision was due to late septic loosening at 3 years post-op. Typical preoperative and postoperative X-ray images demonstrating the technique of complete removal of all acetabular screws are shown in Figs. 3, 4 and 5 illustrates the corresponding images for the technique of partial screw removal.

**Table 1:** Demographic characteristics

Variables	*n* (%)
Male, *n* (%)	9 (64.3)
Left side, *n* (%)	9 (64.3)
Mean age at surgery, years (SD)	64.4 (14.4)
Mean body mass index, kilogram/meter^2^ (SD)	27.4 (8.1)
American Society of Anesthesiologists Physical Status Classification	
I and II, *n* (%)	4 (28.6)
III, *n* (%)	10 (71.4)
Median Charlson comorbidity index (IQR)	2 (2,4)
**Other reasons for revision THA**	
Dislocation/Instability, *n* (%)	1 (7.1)
Loosening femoral stem/peri-stem osteolysis, *n* (%)	6 (42.9)
Massive liner wear, *n* (%)	5 (35.7)
**Surgical approach**	
Anterolateral, *n* (%)	2 (14.3)
Posterolateral, *n* (%)	9 (64.3)
Extended trochanteric osteotomy (ETO), *n* (%)	3 (21.4)
**Revised component(s)**	
Liner only, *n* (%)	1 (7.1)
Liner and head, *n* (%)	4 (28.6)
Liner, head, and stem, *n* (%)	9 (64.3)
**Preop liner types**	
Conventional polyethylene, *n* (%)	11 (78.6)
Highly crosslinked polyethylene (HXLPE), *n* (%)	3 (21.4)
**New liner types**	
Neutral type, *n* (%)	5 (35.7)
Elevated type, *n* (%)	7 (50)
Lateralized-elevated type, *n* (%)	2 (14.3)
**Acetabular component alignment**	
Mean cup inclination, degree (SD)	44.5 (6.6)
Mean cup anteversion, degree (SD)	26.6 (9.2)
Median volume of demineralized bone matrix putty, milliliters (IQR)	10 (10,20)
Median operative time, minutes (IQR)	139 (119,231)
Median intraoperative blood loss, milliliters (IQR)	300 (200,475)
Median length of hospital stays, days (IQR)	3 (3,4)
Median follow-up time, months (IQR)	71.5 (30.5,121.8)

SD: Standard deviation; IQR: Interquartile range

**Table 2:** Implant characteristics and alignment of the cup

Patient	Sex	Age	Side	Cup model	Preop head size	Postop head size	Cup size	Cup inclination	Cup anteversion	Failed Component
1	Male	50	R	HGP2	28	32	(HH)	38	22	No failure
2	Male	83	L	Duraloc	32	36	60	50	20	No failure
3	Male	74	R	Howmedica/osteonic	32	32	58	48	13	No failure
4	Female	56	L	APR Zimmer	28	28	49	36	29	No failure
5	Female	83	L	Duraloc Bantum locking ring	28	36	56	46	37	No failure
6	Female	57	L	Pinnacle ALTRX	36	36	56	42	29	No failure
7	Male	58	L	HGP2	32	32	58	37	39	No failure
8	Female	58	R	P7 Stryker	28	32	66	55	12	No failure
9	Male	79	R	Biomet (Hex lock)	28	36	56	53	31	Aseptic loosening of shell
10	Female	75	L	Pinnacle ALTRX	32	32	52	44	39	No failure
11	Male	69	L	Zimmer Converge Cup	38	38	57	38	21	No failure
12	Male	70	L	Zimmer Trilogy	36	36	64	41	17	No failure
13	Male	32	R	Marathon	28	28	48	54	33	Septic loosening of both components
14	Male	57	L	Osteonics SecurFit	28	32	60	41	31	No failure

There was significant improvement of UCLA, HHS pain, and HOOS JR scores at a median follow-up period of 6 years (Table 3). There were no changes in the VR-12 physical, VR-12 mental, and HHS function scores.

**Table 3:** Patient-reported outcomes

Outcomes	Preop mean	Postop mean	Mean difference (95%CI)	*P-*value
UCLA score	3.3	5.5	1.5 (0.6,2.4)	**0.014**
VR-12 physical subscale	26.0	39.3	13.3 (−8.0,34.5)	0.141
VR-12 mental subscale	46.3	57.0	13.8 (−1.4,28.9)	0.063
HHS pain subscale	12.5	39.7	26.0 (14.7,37.3)	**0.005**
HHS function subscale	24.3	37.3	13.8 (−4.0,31.5)	0.091
HOOS JR	47.0	82.9	34.8 (11.2,58.4)	**0.018**

UCLA: University of California Los Angeles score; VR-12: Veterans RAND 12; HHS: Harris Hip Score; HOOS JR: Hip Disability and Osteoarthritis Outcome Score for Joint Replacement

## Discussion

Retaining a stable acetabular shell during revision hip replacement is a critical decision that offers strong advantages by minimizing surgical trauma and bone loss. When a hip implant fails due to a worn-out liner, but the acetabular cup remains well-fixed, performing a simple liner exchange rather than a full cup revision preserves precious bone stock, reduces blood loss, shortens surgery time, and lowers the risk of major complications like pelvic fractures. This less invasive approach is not always possible, as a poorly positioned cup causing instability must be revised. However, for the growing number of frail, osteoporotic, elderly patients needing revision surgery, the priority shifts from achieving a perfect long-term reconstruction to ensuring patient safety. Bovonratwet et al. demonstrated that aseptic revision THA carries higher risks for patients aged ≥ 80 years compared to younger patients, including increased perioperative mortality, pneumonia, urinary tract infections, greater need for blood transfusions, and longer hospital stays [24]. For octogenarians, retaining the acetabular shell is an effective strategy to minimize surgical risks and promote a safer recovery—a scenario surgeons will encounter more frequently as the population continues to age.

Many methods have been proposed to evaluate polyethylene liner wear [25–27] and help in estimating the need for bone graft/substitute in cases of osteolysis [28, 29]. Cup malposition, instability following screw removal, insufficient bone ingrowth, inability to cement a new liner due to the small cup size or outdated design, and shell erosion by the femoral head are contraindications to retaining the acetabular shell [7, 17, 21, 30]. Ultimately, intraoperative acetabular component testing for stability and positioning is key to planning for isolated liner exchange.

If the cup is retained, injection of bone graft/substitute is challenging due to limited access to the periacetabular bone [18]. Lavernia et al. demonstrated a successful arthroscopic shaver-assisted technique to remove osteolytic lesions proximal to the acetabular cup with limited follow-up of 7 months. In our series, 1 of 14 patients needed reoperation due to loosening of a retained cup after a fall, and another patient had late periprosthetic infection. Our described method is simple, easy, and reproducible, and is a low-cost technique for the delivery of graft material for the management of osteolysis. We recommend consideration of the acetabular shell preservation in the setting of a well-fixed, well-aligned cup with a good track record and available liners, especially in elderly and inactive patients. Ricotti et al. reported a 93.4% revision-free survivorship with only 1.9% radiographic progression at short- to mid-term follow-up after isolated liner exchange and bone grafting for well-fixed cups, recommending this approach for larger lesions (> 450 mm^2^). Our study supports these findings, demonstrating a comparable mid-term survivorship of 85.7% with significant improvements in pain and function using demineralized bone matrix (DBM) delivered via catheter behind a retained, well-fixed shell [31].

No statistically significant differences were observed in some patient-reported outcomes (VR-12 physical subscale, VR-12 mental subscale, and HHS function subscale). The absence of statistically significant improvement in some patient-reported outcomes (VR-12 and HHS function) likely reflects limited statistical power due to the small cohort, potential ceiling effects in a relatively low-demand population, and the clinical trajectory in which pain relief and stability often precede measurable gains in global function after revision THA.

## Limitations

This study has several important limitations. First, the small sample size (*n* = 14) and low event rate (only two failures) limit the statistical power and precision of our effect estimates, which constrains the interpretability of the findings. Second, as a retrospective case series without a control group (e.g., comparing to liner exchange without grafting or full cup revision), the capacity for causal inference and direct comparison is restricted. Third, the applicability of the technique is inherently limited to implants with multiple screw holes, as these provide essential access to the retro-acetabular region. Furthermore, debridement of the inflammatory membrane through these screw holes can be incomplete, potentially compromising both the thoroughness of the debridement and the pressurized delivery of DBM putty into the defect. Fourth, the assessment of graft incorporation was limited because not all patients underwent advanced imaging (CT or MRI). The reliance on radiographs in some cases may have led to an underestimation of osteolysis, potentially biasing the results toward more favorable apparent remodeling.

Finally, to validate these mid-term findings, studies with longer-term follow-up and larger sample sizes are required. Prospective controlled trials—ideally randomized—comparing liner exchange with and without targeted grafting for retro-acetabular osteolysis are warranted to conclusively establish the efficacy of this technique.

## Conclusions

The delivery of demineralized bone matrix via a urine catheter through screw holes is an effective, low-cost technique suited for well-fixed cups with limited osteolysis. It is an option for elderly or low-demand patients, as it minimizes operative time, blood loss, and bone sacrifice.

## Data Availability

The datasets generated during this current study are available from the corresponding author upon reasonable request.

## References

[1] Bayliss LE, Culliford D, Monk AP, Glyn-Jones S, Prieto-Alhambra D, Judge A, et al. The effect of patient age at intervention on risk of implant revision after total replacement of the hip or knee: a population-based cohort study. Lancet. 2017;389(10077):1424–30. 28209371 10.1016/S0140-6736(17)30059-4 PMC5522532

[2] Schreurs BW, Hannink G. Total joint arthroplasty in younger patients: heading for trouble? Lancet. 2017;389(10077):1374–5. 28209372 10.1016/S0140-6736(17)30190-3

[3] Schwartz AM, Farley KX, Guild GN, Bradbury TL. Projections and epidemiology of revision hip and knee arthroplasty in the United States to 2030. J Arthroplasty. 2020;35(6 Suppl):S79-85. 32151524 10.1016/j.arth.2020.02.030 PMC7239745

[4] Kurtz S, Ong K, Lau E, Mowat F, Halpern M. Projections of Primary and Revision Hip and Knee Arthroplasty in the United States from 2005 to 2030: The Journal of Bone & Joint Surgery. 2007 Apr;89(4):780–5. 10.2106/JBJS.F.00222 17403800

[5] Gwam CU, Mistry JB, Mohamed NS, Thomas M, Bigart KC, Mont MA, et al. Current epidemiology of revision total hip arthroplasty in the United States: national inpatient sample 2009 to 2013. J Arthroplasty. 2017;32(7):2088–92. 28336249 10.1016/j.arth.2017.02.046

[6] Siddiqi A, Levine BR, Springer BD. Highlights of the 2021 American Joint Replacement Registry Annual Report. Arthroplast Today. 2022;29(13):205–7. 10.1016/j.artd.2022.01.020 PMC8810304 35128013

[7] Maloney WJ, Paprosky W, Engh CA, Rubash H. Surgical treatment of pelvic osteolysis: clinical orthopaedics and related research. Clin Orthop Relat Res. 2001;393:78–84. 10.1097/00003086-200112000-00009 11764374

[8] Stulberg BN, Della Valle AG. What are the guidelines for the surgical and nonsurgical treatment of periprosthetic osteolysis? J Am Acad Orthop Surg. 2008;16:S20–5. 18612009 10.5435/00124635-200800001-00006

[9] Naudie DDR, Engh CA. Surgical management of polyethylene wear and pelvic osteolysis with modular uncemented acetabular components1. J Arthroplasty. 2004;19(4):124–9. 15190567 10.1016/j.arth.2004.02.019

[10] Callaghan JJ, Hennessy DW, Liu SS, Goetz KE, Heiner AD. Cementing acetabular liners into secure cementless shells for polyethylene wear provides durable mid-term fixation. Clin Orthop Relat Res. 2012;470(11):3142–7. 22585349 10.1007/s11999-012-2380-x PMC3462859

[11] Khanuja HS, Aggarwal A, Hungerford MW, Hungerford DS, Jones LC, Mont MA. Cementing polyethylene liners into non-modular acetabular components in revision total hip arthroplasty. J Orthop Surg (Hong Kong). 2010;18(2):184–8. 20808009 10.1177/230949901001800210

[12] Lim SJ, Lee KH, Park SH, Park YS. Medium-term results of cementation of a highly cross-linked polyethylene liner into a well-fixed acetabular shell in revision hip arthroplasty. J Arthroplasty. 2014;29(3):634–7. 24029718 10.1016/j.arth.2013.07.042

[13] Rivkin G, Kandel L, Qutteineh B, Liebergall M, Mattan Y. Long term results of liner polyethylene cementation technique in revision for peri-acetabular osteolysis. J Arthroplasty. 2015;30(6):1041–3. 25680448 10.1016/j.arth.2015.01.041

[14] D’Antonio JA, Capello WN, Borden LS, Bargar WL, Bierbaum BF, Boettcher WG, et al. Classification and management of acetabular abnormalities in total hip arthroplasty. Clin Orthop Relat Res. 1989;243:126–37. 2721052

[15] Beaulé PE, Leduff MJ, Dorey FJ, Amstutz HC. Fate Of Cementless Acetabular Components Retained During Revision Total Hip Arthroplasty: The Journal of Bone and Joint Surgery-American Volume. 2003;85(12):2288–93. 14668496 10.2106/00004623-200312000-00004

[16] Terefenko KM, Sychterz CJ, Orishimo K, Engh CA. Polyethylene liner exchange for excessive wear and osteolysis. J Arthroplasty. 2002;17(6):798–804. 12216039 10.1054/arth.2002.32705

[17] Maloney WJ, Herzwurm P, Paprosky W, Rubash HE, Engh CA. Treatment of pelvic osteolysis associated with a stable acetabular component inserted without cement as part of a total hip replacement*. The Journal of Bone and Joint Surgery (American Volume). 1997;79(11):1628–34. 9384421 10.2106/00004623-199711000-00003

[18] Narkbunnam R, Amanatullah DF, Electricwala AJ, Huddleston JI, Maloney WJ, Goodman SB. Outcome of 4 surgical treatments for wear and osteolysis of cementless acetabular components. J Arthroplasty. 2017;32(9):2799–805. 28587888 10.1016/j.arth.2017.04.028

[19] Hall A, Eilers M, Hansen R, Robinson BS, Maloney WJ, Paprosky WG, et al. Advances in acetabular reconstruction in revision total hip arthroplasty: maximizing function and outcomes after treatment of periacetabular osteolysis around the well-fixed shell. J Bone Joint Surg Am. 2013;95(18):1709–18. 24048559 10.2106/JBJS.9518icl

[20] Stamenkov R, Neale SD, Kane T, Findlay DM, Taylor DJ, Howie DW. Cemented Liner Exchange With Bone Grafting Halts the Progression of Periacetabular Osteolysis. J Arthroplasty. 2014;29(4):822–6. 24074890 10.1016/j.arth.2013.08.014

[21] Koh KH, Moon YW, Lim SJ, Lee HI, Shim JW, Park YS. Complete acetabular cup revision versus isolated liner exchange for polyethylene wear and osteolysis without loosening in cementless total hip arthroplasty. Arch Orthop Trauma Surg. 2011;131(11):1591–600. 21687959 10.1007/s00402-011-1338-x

[22] Haidukewych GJ. Osteolysis in the well-fixed socket: cup retention or revision? The Journal of Bone and Joint Surgery British volume. 2012;94-B(11_Supple_A):65–9. 10.1302/0301-620X.94B11.30616 23118385

[23] Springer BD, Hanssen AD, Lewallen DG. Cementation of an acetabular liner into a well-fixed acetabular shell during revision total hip arthroplasty. J Arthroplasty. 2003;1(18):126–30. 10.1016/s0883-5403(03)00287-0 14560422

[24] Bovonratwet P, Malpani R, Ottesen TD, Tyagi V, Ondeck NT, Rubin LE, et al. Aseptic revision total hip arthroplasty in the elderly: quantifying the risks for patients over 80 years old. Bone Joint J. 2018;100-B(2):143–51. 29437055 10.1302/0301-620X.100B2.BJJ-2017-0895.R1

[25] Dorr LD, Wan Z. Ten years of experience with porous acetabular components for revision surgery. Clin Orthop Relat Res. 1995;319:191–200. 7554630

[26] Livermore J, Ilstrup D, Morrey B. Effect of femoral head size on wear of the polyethylene acetabular component. J Bone Joint Surg Am. 1990;72(4):518–28. 2324138

[27] Pollock D, Sychterz CJ, Engh CA. A Clinically Practical Method of Manually Assessing Polyethylene Liner Thickness: The Journal of Bone and Joint Surgery-American Volume. 2001;83(12):1803–9. 11741058 10.2106/00004623-200112000-00006

[28] Johanson NA, Driftmier KR, Cerynik DL, Stehman CC. Grading Acetabular Defects: The Need for a Universal and Valid System. J Arthroplasty. 2010;25(3):425–31. 19375888 10.1016/j.arth.2009.02.021

[29] Hozack WJ, Mesa JJ, Carey C, Rothman RH. Relationship between polyethylene wear, pelvic osteolysis, and clinical symptomatology in patients with cementless acetabular components: A framework for decision making. J Arthroplasty. 1996;11(7):769–72. 8934315 10.1016/s0883-5403(96)80175-6

[30] Maloney WJ. Socket retention: staying in place. Orthopedics. 2000;23(9):965–6. 11003101 10.3928/0147-7447-20000901-25

[31] Ricotti RG, Alexander-Malahias M, Ma QL, Jang SJ, Loucas R, Gkiatas I, et al. Isolated Liner Exchange and Bone Grafting for the Management of Periacetabular Osteolysis in Well-Fixed Cups with an Intact Locking Mechanism at Short-Term to Medium-Term Follow-Up: A Systematic Review. HSS Journal®. 2024;20(4):567–76. 10.1177/15563316231189736 PMC11528827 39494435

